# Advantages of Transmuscular Quadratus Lumborum Block via Subfascial Approach Versus Extrafascial Approach for Postoperative Analgesia After Laparoscopic Cholecystectomy: A Randomized Controlled Study

**DOI:** 10.1097/AJP.0000000000001078

**Published:** 2022-10-11

**Authors:** Wen-quan He, Yu-jie Li, Yong-shuai Li, Xu-hao Zhang, Jian Cao, Kai-zhi Lu, Chi Wai Cheung, Jian-teng Gu, Zheng-yuan Xia, Bin Yi

**Affiliations:** * Department of Anaesthesiology, Southwest Hospital, The Third Military Medical University, Chongqing; † Laboratory and Clinical Research Institute for Pain, Department of Anaesthesiology, University of Hong Kong, HKSAR; ‡ Department of Anaesthesiology, Li Ka Shing Faculty of Medicine, The University of Hong Kong, Hong Kong, China

**Keywords:** laparoscopic cholecystectomy, local anesthetics, postoperative analgesia, transmuscular quadratus lumborum block

## Abstract

We aimed to compare the analgesic effect and incidence of lower limb weakness of transmuscular quadratus lumborum (TQL) block via subfascial approach with that via extrafascial after laparoscopic cholecystectomy (LC).

**Methods:**

Eighty patients undergoing LC were randomized to receive ultrasound-guided bilateral TQL block via subfascial (subfascial group) or extrafascial (extrafascial group) using 30 mL of 0.33% ropivacaine unilaterally. Pain scores of port sites while rest and coughing at 1, 6, 12, 24, 36, and 48 hours postoperatively as primary outcome were compared. Modified Lovett Rating Scale, ambulatory dependency, and rescue analgesia requirement was also compared.

**Results:**

The pain score of the subxiphoid and of the right subcostal port site for up to the postoperative 36 hours (2 [1 to 2]) and 24 hours (2 [2 to 3]) in the subfascial group was significantly lower than that in extrafascial group (2 [2 to 2] and 3 [2.25 to 4]). Up to postoperative 24 hours, the rescue analgesia requirement in subfascial group was significantly lower than that in extrafascial group, namely less fentanyl consumption and parecoxib (1.3 [±5.5] μg vs. 5.6 [±10.6] μg; 17.5% vs. 37.5%). The ratio of patients with LRS score of 6 at postoperative 1 hour (65.0%), and with dependent ambulation at postoperative 1 and 6 hours in subfascial group (15.0% and 0.0%) was significantly lower than that in extrafascial group (10.0%, 80.0%, and 17.5%).

**Conclusion:**

TQL block via subfascial had the advantages of better analgesic effect and less lower limbs weakness after LC over that via extrafascial.

Postoperative pain from laparoscopic cholecystectomy (LC) is a mixed of somatic and visceral pain.^1^ The transversus abdominis plane (TAP) block, local infiltration, and/or patient-controlled intravenous analgesia all have been used to reduce postoperative pain.^2^,^3^ The TAP block and local infiltration can reduce the somatic pain, but are ineffective for visceral pain. Patient-controlled intravenous analgesia have been shown to cause nausea, vomiting, or stomach discomfort due to the use of opioid or nonsteroidal anti-inflammatory drugs.^4^

Recently, the transmuscular quadratus lumborum (TQL) block has gained popularity because of its good analgesic efficacy for abdominal surgery^5^–^7^ or hip arthroplasty surgery.^8^–^10^ Kadam et al^6^ reported that the TQL block applied at the dermatomal level covered from T6 to L1 and reduced postoperative pain scores and analgesic consumption after major abdominal surgeries. Ueshima et al^10^ also reported that the TQL block reduced sensation to pinprick in the T11-L4 and T12-L2 dermatomes in the first and second day after surgery, respectively. The blocked dermatomal by the TQL block varied in different clinical studies, and the same phenomena were seen in cadaver studies.^11^–^13^ For example, Adhikary et al^11^ and Carline et al^13^ reported the TQL block on the corpse, and the dye solution mainly distributed to the upper branches of the lumbar plexus in cadavers. However, the cadaver study conducted by Dam et al^12^ suggested that the thoracic sympathetic trunk and the ventral rami of the lower thoracic spinal nerves (T9-T12) were dyed, and the dye solution did not reach the lumbar plexus.

From our clinical experience, we have noted an interesting phenomenon of TQL block that a similar puncture technique and local anesthetic injection position produced a different blocking region when the needle tip placement was slightly different in the quadratus lumborum fascia (subfascia or extrafascia of the anterior thoracolumbar fascia [ATLF]). However, in the existing clinic studies and cadaver studies none of which clearly pointed out the positional relation of the needle tip and the ATLF. Herein, we speculated that the ATLF is a barrier that hinders the local anesthetics spread to the lumbar plexus. If it is true, the dermatomal coverage of subfascial and extrafascial block of ATLF may be different. We conducted this study to test our hypothesis that the ultrasound-guided TQL block with the needle tip placement subfascia (ie, needle tip unpunctured the ATLF) may provide superior analgesia and lower incidence of lower limb weakness for LC when compared with the extrafascia (ie, needle tip punctured the ATLF).

## METHODS

### Patients and Study Design

**FIGURE 1:**
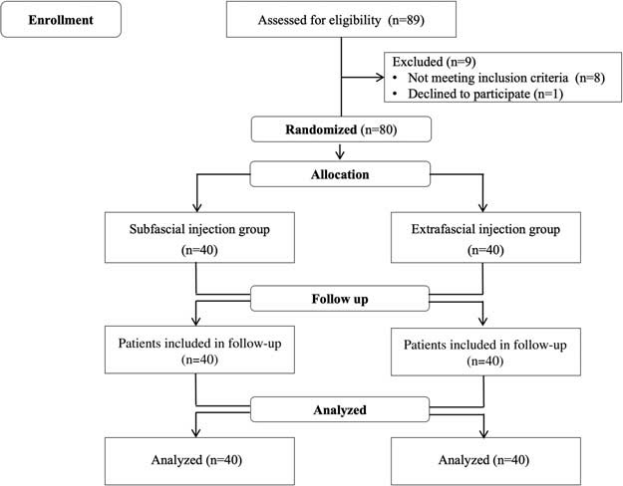
Study flowchart.

The trial protocol was approved by the Ethics Committee of the First Affiliated Hospital of Third Military Medical University (Scientific Research No.16, 2017) and registered at ClinicalTrials.gov (registration NO. NCT03421821, the principle investigator: K.-z.L.) on January 22, 2018. We did not enroll any patients until we completed the registration of the trial on line. Written informed consent was obtained from each enrolled patient. Between February 25 to September 17, 2018, patients scheduled for elective LC surgery with American Society of Anesthesiologists physical status I and II, age 18 to 75 years old, body mass index of 17 to 32 kg/m^2^, and with operation time <2 hours were enrolled (Fig. 1). All patients with a history of local anesthetic allergy, chronic opioid usage, or communicative disorders were excluded from the study. Patients were randomly allocated to either the subfascial group (needle tip unpunctured the ATLF) or the extrafascial group (needle tip punctured the ATLF) using a random number table. The investigator who took charge of patient recruitment, allocation, and quality control did not participate in perioperative anesthesia and pain management. The quadratus lumbar block and general anesthesia were conducted by the same experienced anesthetist. Postoperative pain management, related evaluation, follow-up, and data collection were conducted by another doctor who was not involved with treatment of the patients (the researcher). The researcher was blinded with the study protocol, independently harvested the all above outcome measures.

### Preanesthesia and Ultrasound-guided TQL Block

All patients received routine monitoring, for example, electrocardiogram, noninvasive blood pressure, oxygen saturation, end-tidal carbon dioxide, and Bispectral Index, and perioperative anesthesia management. After arriving in the operating room, a 16-G intravenous cannula was inserted in the right hand or arm.

Patients in the lateral position, after skin disinfection with iodophor disinfection solution, a low-frequency broadband (5 to 8 MHz) convex probe (Shenzhen Wisonic Co. Ltd) was placed transverse to the flank between the lower costal margin and the iliac crest to provide a view of the Shamrock sign.^14^ A 110-mm insulated needle (B. Braun Melsungen AG) was introduced in the plane of the ultrasound beam and advanced until it reached the place between the ATLF and the quadratus lumborum or the psoas major. The needle tip was advanced to the ATLF, and the optimal point of injection, the needle tip either not puncturing (subfascial group) or puncturing (extrafascial group) the ATLF, was determined using hydrodissection. Then, the anesthetist injected 0.33% ropivacaine 30 mL in the either extrafascial or subfascial after confirming negative aspiration. The same method was applied to the opposite side to perform TQL block. The patients were closely monitored for 30 minutes after performing the block. The sensory level was assessed by the researcher, who was blinded with the study protocol, with cold sensation (ice cube) in each dermatomal distribution from T4 to L5 every 5 minutes for 4 times.

### General Anesthesia and Surgery

After preoxygenation, the induction of anesthesia was achieved with intravenous etomidate (0.3 mg/kg), cisatracurium besilatet (0.2 mg/kg), and sufentanil (0.4 μg/kg). Then, an endotracheal tube was inserted, and mechanical ventilation was started. General anesthesia was maintained with the target-controlled infusion of propofol (2.5 to 3 μg/mL) and remifentanil (2.5 to 3 ng/mL) and the depth of anesthesia was monitored with the Bispectral Index. Hydroxyethyl Starch 130/0.4 and normal saline were used for fluid management perioperatively. After completion of the surgery, all patients were transferred to the postanaesthesia care unit and then extubated before returned to the surgical ward.

In our hospital, the hepatobiliary surgeons routinely choose the subxiphoid, right subcostal, and supraumbilical incision as the trocar puncture holes for performing LC. We evaluated the efficacy of subfascial or extrafascial block in LC postoperative analgesia by a 3-point method (the independent Visual Analog Scale [VAS] score of the subxiphoid, subcostal, and supraumbilical incision). The method was more suitable to the evaluate the pain area of post-LC.^15^ Three surgeons each with at least 10-year clinical experience, took part in the clinical trial. All patients stayed in the hospital during the study period.

### Study Endpoints and Rescue Analgesia

The primary outcome was the pain scores of the subxiphoid, subcostal, and superumbilical port sites after surgery. Postoperative pain at rest and with cough were assessed using the VAS pain scores (VAS 0 to 10, 0=no pain; 10=pain as bad as can be) at 1, 6, 12, 24, 36, and 48 hours after the surgery. Secondary outcomes were modified Lovett Rating Scale (LRS), sensory level, proportion of dependent ambulation, parecoxib sodium and fentanyl consumption, adverse effects, and patient satisfaction. The modified LRS was assessed at 30 minutes after TQL block and it was defined as follows: 0=complete paralysis; 1=almost complete paralysis; 2=pronounced mobility impairment; 3=slightly impaired mobility; 4=pronounced reduction of muscular force; 5=slightly reduced muscular force; 6=normal muscular force. The proportion of dependent ambulation was recorded at 1 and 6 hours after surgery. Patient satisfaction was assessed using a scale of 0 to 10, 10 being the most satisfied, at postoperative 48 hours. Any adverse effects were recorded, included postoperative nausea and vomiting, pruritus, gastroduodenal ulcer, and local anesthetics toxicity. Any complications through hospital stay were recorded. The researchers, who was blinded with the study protocol, independently collected all outcome measures.

Postoperative acute pain management program was implemented in throughout our study. Parecoxib sodium was administered with 40 mg intravenous infusion when the patient’s resting VAS was >5 points. After 20 minutes, if pain was not effectively controlled, 25 μg of fentanyl was administered when necessary until the pain was relieved. No additional parecoxib sodium was given for 6 hours, and the total amount was not >80 mg within 24 hours.

### Sample Size Calculation

In our routine work, patients who got the rescue analgesia were carefully recorded. When calculating the sample size, we retrospectively analyzed the incidence of fentanyl for rescue analgesia at the postoperative 6 hours with TQL block (the failure of postoperative analgesia) and found that the incidence was up to 65%. So we assumed that if the subfascial block of ATLF would reduce the incidence to 30%, the subfascial block was superior. When we set at an α error of 0.05 and a power of 80%, a minimum of 31 patients in each arm was needed.

### Statistical Analysis

Normally distributed continuous data were analyzed using unpaired Student *t* tests. VAS scores were analyzed with Mann-Whitney *U* tests. Nausea and vomiting incidence, analgesic consumption, and lower limb strength were analyzed with χ^2^ tests. Categorical data were analyzed with Fisher exact tests. Accordingly, data were presented as mean (SD), median (M), and interquartile range, percentage of population, or box-whiskers plot wherever appropriate. A significance level for all analyses was set a *P* value <0.05. Statistical analysis was performed using SPSS (version 22.0; IBM Corp.).

## RESULTS

A total of 89 patients who underwent LC were recruited to the study (Fig. 1). The surgery time for 3 patients from the subfascial group and 4 patients from the extrafascial group was >2 hours, and they were excluded for data analysis. One patient refused to participate the study. Thus, 80 patients underwent randomization and were assigned to the subfascial or the extrafascial group equally. There were no significant differences of patients’ characteristics between the 2 groups (Supplementary Table I, Supplemental Digital Content 1, http://links.lww.com/CJP/A895).

### Ultrasound Images

**FIGURE 2:**
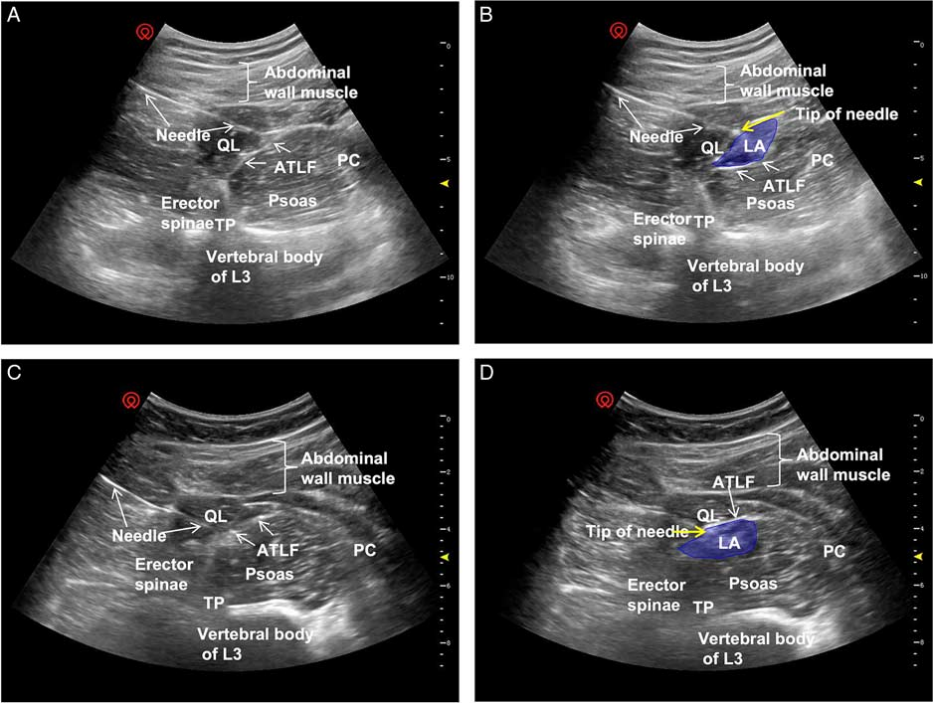
Ultrasound images of the TQL block approach. A and C, Images of the desired sonographic landmarks were visualized, including the QL muscle, erector spinae, psoas muscle, TP, ATLF, and PC. B, Image of the ATLF subfascial injection of local anesthetics (blue). The QL was pushed toward the probe by the local anesthetics, and the morphology of the psoas was almost unchanged. D, Image of the ATLF extrafascial injection of local anesthetics (blue). The psoas was pushed toward the vertebral body by the local anesthetics, and the morphology of the QL was almost unchanged. ATLF indicates anterior thoracolumbar fascia; PC, peritoneal cavity; QL, quadratus lumborum; TP, transverse process; TQL, transmuscular quadratus lumborum.

In the ultrasound images of the subfascial group, the local anesthetics solution forms a fusiform hypoechoic image between the ATLF and the quadratus lumborum was found, and the quadratus lumborum was pushed toward the probe by the local anesthetics solution. In the extrafascial group, the local anesthetics solution formed a fusiform hypoechoic image between the ATLF and the psoas major was noted, and the psoas major was pushed toward the vertebral body by the local anesthetics solution (Fig. 2).

### Pain Scores and Analgesic Consumption

**FIGURE 3:**
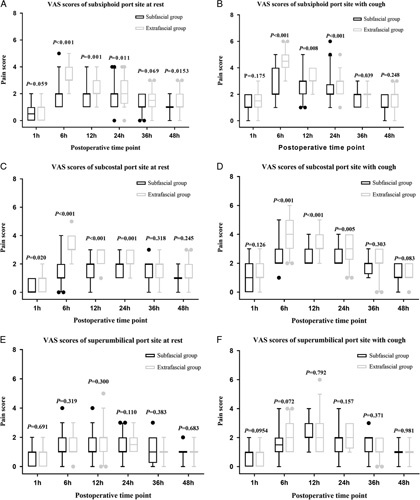
Pain scores (as assessed with the VAS for pain) at rest and during coughing for the 3 port sites (subxiphoid, subcostal, and supraumbilical port site) in both groups during the first postoperative 48 hours. VAS scores of subxiphoid port site at rest (A) and with cough (B). VAS scores of subcostal port site at rest (C) and with cough (D). VAS scores of supraumbilical port site at rest (E) and with cough (F). Data are presented as box-and-whisker plots. The line across the box indicates the median, top, and bottom of the box represent the 25th and 75th percentile values, error bars represent 5% and 95% values, and circles represent outlier values. Differences in VAS scores at each time point between the 2 groups were analyzed using the Mann-Whitney *U* test. VAS indicates Visual Analog Scale.

The VAS pain score of the subxiphoid port site in the subfascial group were significantly less than that of the extrafascial group at the postoperative at 6 hours (2 [1, 2] vs. 3 [3, 4], *P*<0.001), 12 hours (2 [1, 2] vs. 2 [2, 3], *P*=0.001), and 24 hours (2 [1, 2] vs. 2 [1.25, 3], *P*=0.011) at rest. And 6 hours (2 [2, 4] vs. 4.5 [4, 5], *P*<0.001), 12 hours (3 [2, 3] vs. 3 [3, 4], *P*=0.008), 24 hours (2 [2, 2.75] vs. 3 [2, 3], *P*<0.001), and 36 hours (2 [1, 2] vs. 2 [2, 2], *P*=0.039) postoperatively with cough (Figs. 3A, B). The VAS pain score of the right subcostal port site in the subfascial group were significantly lower than in the extrafascial group at the postoperative at 1 hour (0 [0, 1] vs. 1 [0, 1], *P*=0.02), 6 hours (1 [1, 2] vs. 3 [3,4], *P*<0.001), 12 hours (2 [1, 2] vs. 3 [2, 3], *P*<0.001), and 24 hours (2 [1, 2] vs. 2 [2, 3], *P*=0.001) at rest and 6 hours (2 [2, 3] vs. 4 [3, 5], *P*<0.001), 12 hours (2 [2, 3] vs. 3 [3, 4], *P*<0.001), and 24 hours (2 [2, 3] vs. 3 [2.25, 4], *P*=0.005) (Figs. 3C, D). The VAS pain score of the superumbilical port site at rest and during coughing were not significantly different (Figs. 3E, F).

**FIGURE 4:**
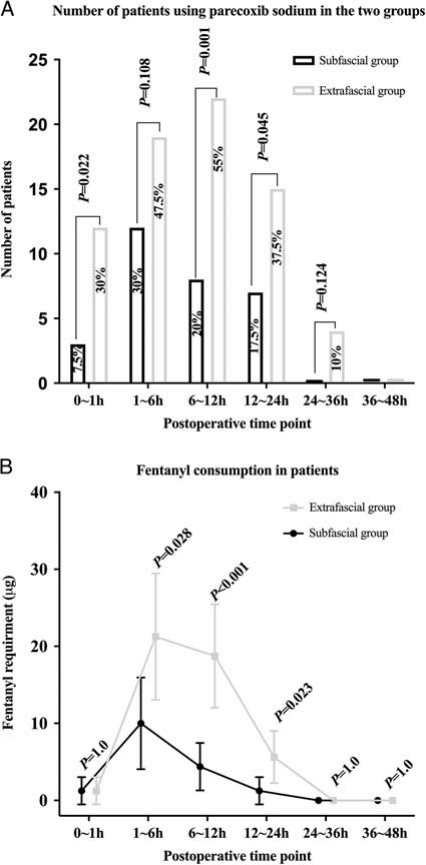
The number of patients using parecoxib sodium (A) and fentanyl consumption in the patients (B) receiving transmuscular quadratus lumborum block through subfascial or extrafascial injection at different postoperative time points. A, Percentage of patients with parecoxib sodium consumption during the different postoperative period. B, Fentanyl consumption in the different postoperative period. Data are expressed as the means with 95% CIs.

Compared with the extrafascial group, the subfascial group had significant fewer patients who received parecoxib sodium at 1 hour (7.5% vs. 30%, *P*=0.022), 12 hours (20% vs. 55%, *P*=0.001) and 24 hours (17.5% vs. 37.5%, *P*=0.045) after surgery (Fig. 4A). In addition, the fentanyl consumption was less at 6 hours (10.0 [18.6] μg vs. 21.3 [25.7] μg, *P*=0.028), 12 hours (4.4 [9.6] μg vs. 18.8 [21.0] μg, *P*<0.001), and 24 hours (1.3 [5.5] μg vs. 5.6 [10.6] μg, *P*=0.023) after surgery in the subfascial group (Fig. 4B).

### Sensory Block Level and Lower Limb Muscle Strength

**FIGURE 5:**
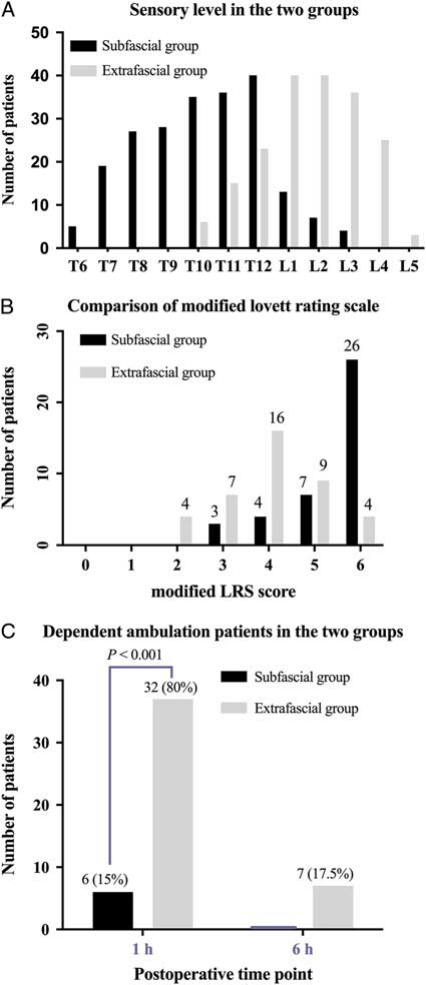
Blocking effectiveness of transmuscular quadratus lumborum block *via* subfascial superior over extrafascial approach. A, Sensory level distribution in the 2 groups. Data are presented as the number. B, The modified LRS after 1 hour surgery in the 2 groups. Data are presented as the number. C, The proportion of dependent ambulation assistance at 1 and 6 hours after surgery in the 2 groups. Data are presented as the number or percent. The χ^2^ tests were used for intergroup comparisons. In the subfascial group, the proportion of dependent ambulation at 1 and 6 hours after surgery and LRS scores were significantly higher when compared with the extrafascial group, *P*<0.001. LRS indicates Lovett Rating Scale.

In the subfascial group, the cutaneous sensory block region was from T6 to L4 but the majority was from T7-T8 to T12-L1. In the extrafascial group, the cutaneous sensory block region was from T10 to L5 but mainly was from T11-T12 to L3-L4 (Fig. 5A).

We compared the postoperative lower limb muscle weakness by the LRS and ambulatory dependency. For the distribution of LRS in the 2 groups, that in subfascial group achieved higher LRS. More specifically, the ratio of patients with normal muscular force (a LRS score of 6) in the subfascial group was significantly higher than that in the extrafascial group (65% vs. 10%, *P*<0.05; Fig. 5B). In addition, the ratio of patients with more than pronounced reduction of muscular force (LRS ≤4) in the subfascial group was significantly less than that in the extrafascail group (17.5% vs. 67.5%*, P*<0.001; Fig. 5B). Moreover, the ratio of patients who need dependent ambulation in the subfascial group at 1 and 6 hours postoperatively was significantly lower than that in the extrafascial group (1 h:15% vs. 80%, *P*<0.001; 6 h: 0 vs. 17.5%, *P*<0.001), respectively (Fig. 5C).

### Adverse Effect and Patient Satisfaction

There was no statistically significant difference between the 2 groups in the incidence of nausea and vomiting or in pruritus (*P*>0.05). There were 2 patients and 4 patients with postoperative dysuria in the subfascial group and the extrafascial group, respectively, but the difference was not significant (*P*=0.675). There were no cases of gastroduodenal ulcer or local anesthetics intoxication in the 2 groups. There was no significant difference in patient satisfaction between the 2 groups (Supplementary Table II, Supplemental Digital Content 1, http://links.lww.com/CJP/A895, *P*=0.102).

## DISCUSSION

Our data suggested that ultrasound-guided TQL block via subfascial provided superior analgesia, while reduced the incidence of lower limb weakness in the LC when compared with that via extrafascial. The current study also showed that TQL block via subfascial for LC reduced the consumption of parecoxib sodium and fentanyl compared with that via extrafascial.

To date, 4 techniques of quadratus lumborum block have been used:^16^–^19^ (1) the quadratus lumborum block 1 or lateral quadratus lumborum block, injecting the local anesthetics at the anterolateral aspect of the quadratus lumbar; (2) the quadratus lumborum block 2 or posterior quadratus lumborum block, injecting the local anesthetics at the posterolateral aspect of the quadratus lumbar; (3) the quadratus lumborum block 3 or TQL block or anterior quadratus lumborum block, injecting the local anesthetics between the quadratus lumbar and psoas major; (4) the intramuscular quadratus lumborum block, injecting the local anesthetics in the quadratus lumborum. Among those techniques, the position of the needle tip and the ATLF has not been clarified in those previous studies.^16^,^17^,^19^ The “best” needle tip position has not been established yet and its position might affect the region of local anesthetic diffusion. For example, the inconsistent data showed that the fascia iliaca block resulted from relatively minor variations in needle position.^20^ From this finding, we speculated that the inconsistency in the TQL blockade area might be similar to that of fascial iliaca blockade and the ATLF play an important role in the phenomenon.

**FIGURE 6:**
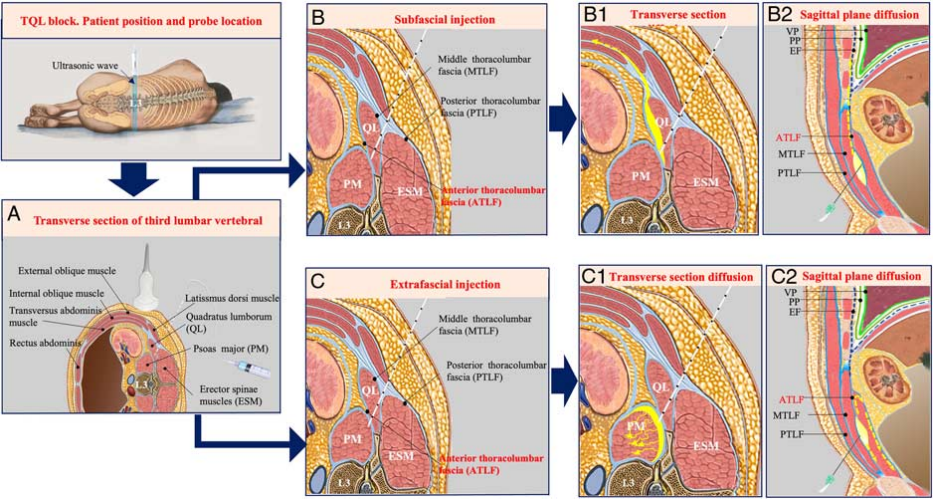
Anatomic schematic illustration representing the ultrasound TQL block (A) and the distribution of local anesthetics (yellow) during a subfascial injection (B: B-1 and B-2) or extrafascial injection (C: C-1 and C-2). B-1, After the subfascial injection, the local anesthetics spread through the ATLF to the endothoracic fascial to reach the subendothoracic space, a schematic of the sagittal plane. B-2, After the subfascial injection, the local anesthetics spread to the transversus abdominis plane, a schematic of the transverse section. C, Extrafascial injection schematic. C-1, After the extrabfascial injection, the local anesthetics spread along the ATLF and little local anesthetics across the 12st rib, a schematic of the sagittal plane. C-2, After the extrafascial injection, the local anesthetics diffused along the potential gap between the ATLF and the psoas major, a schematic of the transverse section. “Yellow arrow” the direction of local anesthetics diffusion. ATLF indicates anterior thoracolumbar fascia; EF, endothoracic fascia; PP, parietal pleura; TQL, transmuscular quadratus lumborum; VP, visceral pleura.

In the subfascial block, in which the insulated needle tip does not puncture the ATLF, after injecting the local anesthetics between the ATLF and the quadratus lumborum, local anesthetics diffuse along the ATLF to the endothoracic fascia and reach the subendothoracic space to produce lower thoracic nerve block. Because the ATLF was formed by the medial continuation of the transversalis fascia and the investing fascia of the psoas,^21^ the transversalis fascia was continuous with the endothoracic fascia,^22^ and the subendothoracic space communicated with the lower thoracic paravertebral space.^23^ According to this, local anesthetics eventually spread along the fascia plane to the lower thoracic paravertebral space, the transversalis fascia plane and the transversus abdominis plane, resulting in blockage in the widespread abdominal region. On the other hand, the ATLF may act as a barrier, impeding the spread of some local anesthetics to the lumbar plexus and reducing the possibility of lumbar plexus block (Figs. 6A, B: B-1 and B-2). Therefore, in our trial, the subfascial approach produced reliable sensory level mainly covering from T7-T8 to T12-L1, and the pain scores of the subxiphoid and subcostal port sites were much lower than that in the extrafascial group.

In the extrafascial block, the insulated needle tip punctures the ATLF; after injecting the local anesthetics between the ATLF and psoas major and then the local anesthetics would diffuse along the potential gap between the ATLF and the psoas major, and reach the lumbar paravertebral region to block upper branches of the lumbar plexus.^11^ Moreover, the psoas muscle, while housing the lumbosacral plexus, may be commonly split by a fascial layer between the posterior one third and anterior two third of the muscle,^24^ the local anesthetic would spread along the fascial and psoas muscle bundle, infiltrating part of the lumbar plexus, resulting in some patients’ lower extremity weakness. (Figs. 6A, C: C-1 and C-2). In the reports of La Colla et al^25^ and Sondekoppam et al,^26^ the quadratus lumborum block provides postoperative analgesia for total hip arthroplasty and iliac crest bone graft harvesting surgery, and the mechanism may be attributed to extrafascial block to cause patient’s lower limb was weak. In our trial, the extrafascial group produced sensory level from T11-T12 to L3-L4, the sensory paralyses region could satisfy the requirement of postoperative analgesia for hip surgery. But the probabilities of lower limb weakness proportion and patients needed dependent ambulation for 6 hours after surgery were significantly higher than in the subfascial group. It should be pointed out that in some obese patients, due to the poor quality of ultrasound images, we could not exactly identify the location of the needle tip and ATLF, leading to intramuscular (quadratus lumborum or psoas major) injection or extrafascial injection. In those patients, subfascial injection with the subcostal TQL block technique^21^ may improve the quality of ultrasound images to identify the ATLF. In addition, when the exactly ATLF cannot be identified, intramuscular quadratus lumborum block was done instead of piercing ATLF to extrafascial or intramuscular psoas major injection. In this case, the analgesic effect is reduced, but the weakness of the lower limbs can be avoided.

Effective postoperative analgesia, minimally invasive surgery, and early postoperative mobility were the key components of enhanced recovery after surgery.^27^ Weakness in the lower limbs prolongs bed rest time, which is not conducive to early recovery of gastrointestinal function and might also increase the risk of deep vein thrombosis.^28^,^29^ Quadratus lumbar block was considered an indirect thoracic paravertebral block (TPVB).^12^,^30^ Meanwhile, the thoracolumbar fascia with high density of sympathetic neurons^31^ was considered another major factor responsible for the effects of quadratus lumbar block. Therefore, allowing more local anesthetics spreading to the TPVB and TLF can produce better visceral and incision analgesia. In this theory, subfascial block allowed more local anesthetics spreading to the TPVB and to deposit on the ATLF to block more sympathetic neurons. Therefore, the implications of our study may be that the novel block reported herein may contribute to enhanced recovery after surgery if it could be used widely in clinic practice.

There were several limitations in the current study. First of all, there is some limitation related to the techniques and equipment used in this single-center study, the application of this technics to different levels of medical centers needs further efforts. Second, this is a single center and small sample size research, though we tried our best to reduce bias, multicenter research should be conducted to validate our results.

## CONCLUSIONS

Our study demonstrated that the sensory region by the TQL block via subfascial was from T7-T8 to T12-L1, while the cutaneous sensory block region mainly was from T11-T12 to L3-L4 by that via extrafascial. The VAS score of pain, the demand of analgesics, and the incidence of lower limb weakness after the operation were significantly lower in the TQL block via subfascial than that via extrafascial, and the incidence of lower limb weakness was lower. Our data suggested that ultrasound-guided TQL block via subfascial approach should be considered to use clinically but warrants further study.

## Supplementary Material

SUPPLEMENTARY MATERIAL
