# Supplementary material for: Advantages of Transmuscular Quadratus Lumborum Block via Subfascial Approach Versus Extrafascial Approach for Postoperative Analgesia After Laparoscopic Cholecystectomy: A Randomized Controlled Study
Source: Clin J Pain. 2022 Oct 11;38(12):730–8. doi: 10.1097/AJP.0000000000001078 (PMC9645534; doi:10.1097/AJP.0000000000001078)
Supplement: SUPPLEMENTARY MATERIAL [file ajp-38-730-s001.docx]

**Supplementary Table Ⅰ**. Patient characteristics. Patient characters and clinical data presented as mean (95% confidence interval) or absolute number as appropriate. All variables were similar between the two groups.

|  | Subfascial group | Extrafascial group | *P* value |
| --- | --- | --- | --- |
| Sample size, n | 40 | 40 |  |
| Age (years) | 46.5±11.1 | 43.3±12.3 | 0.222 |
| Sex (male/female) | 27/13 | 30/10 | 0.621 |
| ASA physical status (I/II) | 19/21 | 16/24 | 0.652 |
| Weight (Kg) | 62.3±10.0 | 62.7±10.9 | 0.865 |
| Height (cm) | 160.4±7.6 | 161.2±12.3 | 0.630 |
| Body mass index (kg·m^-2^) | 24.2±3.1 | 24.1±3.4 | 0.882 |
| Duration of surgery (min) | 54.9±19.0 | 51.0±16.5 | 0.321 |

Patient characters and clinical data presented as mean (95% confidence interval) or absolute number as appropriate. All variables were similar between the two groups.

**Supplementary Table Ⅱ. Adverse effects and patient satisfaction.**

|  | Subfascial group | Extrafascial group | *P* Value |
| --- | --- | --- | --- |
| Sample size, n | 40 | 40 |  |
| PONV, n (%) | 6 (15) | 7 (17.5) | 0.762 |
| Pruritus, n (%) | 0 | 0 | 1.0 |
| Gastroduodenal ulcer, n (%) | 0 | 0 | 1.0 |
| LA toxicity, n (%) | 0 | 0 | 1.0 |
| Postoperative dysuria, n (%) | 2 (5) | 4 (10) | 0.675 |
| Patient satisfaction, n (%) | 9.0 (±1.0)  9 [8,10] | 8.6 (±1.1)  9 [8,9] | 0.102 |

Patients interviewed at 48 h using a scale of 0–10 with 10 being the most satisfied. Patient satisfaction data reported as the mean (± SD) and median [interquartile range]. Chi-square tests or Fisher exact tests were performed to compare the incidence of side effects. Patient satisfaction was analyzed using Mann-Whitney U-tests. *P*<0.05 is considered significant. PONV, postoperative nausea and vomiting, LA, local anaesthetics, PO=postoperative
